# Effects of Space Flight on the Chemical Constituents and Anti-Inflammatory Activity of Licorice (*Glycyrrhiza uralensis *Fisch)

**Published:** 2012

**Authors:** Jingze Zhang, Wenyuan Gao, Shuo Yan, Yaxin Zhao

**Affiliations:** *School of Pharmaceutical Sciences and Technology, Tianjin University, Tianjin 300072, China.*

**Keywords:** Licorice, Space flight, HPLC, Anti-inflammatory effect

## Abstract

Licorice, the oldest Chinese traditional medicine, is widely used in the treatment of human diseases. Due to the deficiency of wild resource, selecting and breeding becomes a key issue to expanding the supply of licorice. Spaceflight technology will become a new method for medicinal plants. The aim of this study was to investigate the effect of spaceflight on the components and anti-inflammatory activity in licorice. After flowing on a recoverable satellite for 18 days, licorice seeds were germinated and grown to maturity and the parallel ground-based seeds were also planted under the same conditions. The main components in licorice root were analyzed through HPLC. The contents of two components in spaceflight groups were higher than that of the ground control ones. Three acute inflammatory models including xylene-induced auricular edema, carrageenan-induced paw edema and acetic acid-induced vascular permeability were utilized to compare the anti-inflammatory activity of licorice pre and post spaceflight. The licorice extract showed the significant anti-inflammation activity. After the spaceflight, the pharmacological activity of licorice got higher than that of the ground control one. All of the models gained the tendency that the spaceflight group of species Hangjinqi had the strongest activity than other groups. The research provided the scientific data for a new breeding of medicinal plant through the spaceflight and indicated that the technology of space flight may be a new effective method for the breeding and cultivation of licorice.

## Introduction

Licorice, derived from the dried roots and rhizomes of *Glycyrrhiza* species (Leguminosae family), is one of the oldest and most commonly used Chinese traditional medicines (TCM) in China (1). Clinical and experimental studies show a variety of pharmacological activities of *Glycyrrhiza uralensis *including anti-inflammatory, antioxidative, antiviral, antiulceric, antispasmodic, antiallergic, antidiabetic, anticancer, hepatoprotective, expectorant and memory enhancing activities, *etc* (2-6). In addition to its sweet taste, licorice is also widely utilized as flavoring additives in soft drinks, candies and other foods. Therefore, here is a great demand for it in food industry. There are about 30 species containing in *Glycyrrhiza *genus all over the world. According to Chinese Pharmacopoeia, only three species including *Glycyrrhiza uralensis, G. glabra*, *and G. inﬂata* are officially used as licorice. Traditionally, this medicinal plant was collected from wild growing populations. However, over-collection of wild licorice induced a rapid decrease in wild resources and led to desertification of the habitat. Selecting and breeding becomes a key issue to expanding the medical resource of licorice. However, the low germination rate and content of active components in cultivated licorice limit its large-scale cultivation (7-8). High radiation, microgravity and other space factors may change the hereditary in plants and create a new breed (9-10).

Licorice seeds were flown on a recoverable satellite for 18 days. After returning to earth, the seeds were germinated and grown to maturity. The parallel ground-based seeds were also planted under the same conditions. In our previous work, as shown in results, after the spaceflight the seeds not only had high soluble protein and peroxidase (POD) and catalase (CAT) activity but also its germination had many different characters compared to the control ground group (11-12). In the inter-simple sequence repeat analysis, among 22 random primers used, 6 primers generated different DNA band types.

The main chemical constituents may significantly vary due to many factors such as the different plant species, geographic sources, harvesting and processing, and thus, affect the pharmacological activity of licorice. In the recent years, several recoverable satellites have been launched and the researches about the effects of spaceflight mainly focused on the diversities of growth, development, physiological and genetic characteristics of the plants. Heretofore, the study on comparison and identification of multiple components between the spaceflight plant and the ground control sample via high performance liquid chromatography (HPLC) has been hardly reported. Furthermore, it is unknown whether the spaceflight can influence the pharmacological activity of the medicinal plant. The aim of the present study was to utilize the HPLC method to identify the main components in licorice and compare the differences pre and post spaceflight and the anti-inflammatory activity was compared between the samples. To the best of our knowledge, it was the first time to report that series of changes were studied focusing on the main components and pharmacological activity of licorice pre and post spaceflight. The research will provide the scientific data for a new breeding of medicinal plant by spaceflight.

## Experimental

*Plant material*

Wild licorice (*Glycyrrhiza uralensis *Fisch.) seeds were obtained from Inner Mongolia Autonomous Region in China. ACK, which represented the ground control samples, was the seeds from Ordos, Inner Mongolia. HCK, which represented the ground control samples, was the seeds from Hangjinqi, Inner Mongolia. Some wild licorice seeds from the group of ACK and HCK were glued to a board to form a seed biostack and then loaded into a small, hermetically sealed biocabin constructed of an all-sealed aluminum alloy.

The biocabin, which contained a self-regulating oxygen generator that used KO_2_ and LiOH, was carried using the 18^th^ recoverable satellite for 18 days. Average radiation dose in the flight recovery module was 0.102 mGy day^-1^ (mGyday^-1^ means the unit of absorbed radiation dose of ionizing radiation in one day and g is the unit of gravity). The distance from flight apogee to earth was and the gravity was 10^−6^×g. The temperature in the cabin fluctuated between 7.5 and according to the relative location of the satellite to the sun during the period of running. Sample A and H were represented the spaceflight group of ACK and HCK respectively. During the spaceflight, the ground control seeds were placed in an incubator remained on the earth at . After the spaceflight, all of the seeds from the ACK, HCK, A and H groups were sown and cultivated under the same condition in Jiangsu Minqin experiment base. With an adequate water supply, they grew in a greenhouse at , under lights (2,000-3,000 µmol m^-1^s^-1^). One-year-old roots were collected and identified according to the botanical morphology of *Glycyrrhiza uralensis *Fisch, characteristic and microscopic character description of the roots recorded in Chinese Pharmacopoeia.

*Chemical reagents*

HPLC grade acetonitrile was purchased from Fisher (USA). Water was purified with a Milli-Q water purification system (Millipore, USA). Methanol and acetic acid were of analytical grade and were purchased from Guangfu Technology Limited Company (Tianjin, China).

Liquiritin and glycyrrhizic acid were purchased from the National Institute for the Control of Pharmaceutical and Biological Products (Beijing, China). The purity of each compound was determined to be higher than 98% through HPLC.

Xylene was purchased from Yingdaxigui Chemical Industry (Tianjin, China). Glacial acetic acid was purchased from Guangfu Technology Limited Company (Tianjin, China). Evan’s blue was purchased from Sigma Industries (St. Louis, MO, USA). Dexamethasone was purchased from Lisheng Medical Limited Company (Tianjin, China).

*Animals*

Kunming mice (♀), weighing about 18-22 g; Sprague-Dawley rats (♀), weighing about 180-220 g were used in the experiments. The animals were purchased from the Experimental Animal Center, Chinese Academy of Medical Sciences, Peking, SCXK-2007-004. The animals were housed in an environmentally (t = 25°C) and air humidity (60%) controlled room with a 12-h light-dark (07:00-19:00 h and 19:00-07:00 h) cycle, kept on a standard laboratory diet and drinking water ad-libitum. This study was carried out in accordance with the “Regulation for the Administration of Affairs Concerning Experimental Animals (State Council of China, 1988).

*Preparation of licorice root extracts*

One-year-old roots of different samples (A; ACK; H and HCK) were pulverized into powder and were subjected to extraction with 10 times of distilled water, three times, 2 h each. The aqueous extracts were evaporated (2 Kg/L) and stored in the freezer (-4°C).

*HPLC-DAD analysis*

All licorice root extracts were analyzed through an Agilent 1100 liquid chromatograph system (Agilent Technologies, USA), consisting of a quaternary pump, an online degasser, and a column temperature controller, coupled with a photodiode array detector. Separations were carried out with a C_18_ Kromasil column (250 mm×4.6 mm ID, 5 µm) and the column temperature was kept at 35°C. The mobile phase was a linear gradient prepared from water (containing 1% acetic acid) (A) and acetonitrile (B). The composition of the gradient was A:B, 90:10 at 0 min, 84:16 at 10 min, 81:19 at25 min, 40:60 at 97 min, 40:60 at 107 min and then the system was returned to initial conditions. The flow rate was 1 mL/min, and the injection volume was 20 μL.

*Xylene-induced auricular edema*

Mice were divided into 14 groups (10/group). In group 1, animals (control) received normal saline whereas in groups 2, animals were treated with dexamethasone (DEX, 10 mg/Kg). In group 3 to 5 animals received sample extract A (1, 2, 4 g/Kg); group 6 to 8 animals received sample extract ACK (1, 2, 4 g/Kg); group 9 to 11 animals received sample extract H (1, 2, 4 g/kg); and group 12 to 14 animals received sample extract HCK (1, 2, 4 g/Kg). Successive administration was used for 5 days. At the fifth day, 45 min after *i.g.* administration of the tested fraction, 0.1 mL of xylene was applied to the anterior and posterior surface of the ear of each mouse. The left ear was considered as a control. One and half hour after the xylene application, the mice were killed (cervical dislocation) and both ears were taken. Circular sections were taken using a cork borer with a diameter of 8 mm, and weighed. Oedema was assessed in terms of the mean weight increase of each ear, while the inhibition of Oedema was expressed as the weight reduction in comparison with the control group (13).

*Carrageenan-induced paw edema*

Rats were divided into 14 groups (10/group). Group 1 animals (control) received the normal saline whereas group 2 animals were treated with dexamethasone (DEX, 10 mg/Kg). In group 3 to 5, animals received sample extract A (0.5, 1, 2 g/Kg); group 6 to 8 animals received sample extract ACK (0.5, 1, 2 g/Kg); in group 9 to 11 animals received sample extract H (0.5, 1, 2 g/Kg); and group 12 to 14 animals received sample extract HCK (0.5, 1, 2 g/Kg). Successive administration was used for 5 days. At the fifth day, before any treatment, paw thickness was measured from ventral to dorsal surfaces on the right paw of each animal with a dial caliper, immediately prior to carrageenan injection. Test materials were administered perorally and 45 min later freshly prepared carrageenan (0.1 mL of 1% suspension in normal saline) was injected in the sub-plantar region of the right hind paw. The paw thickness was measured at 0.5, 1, 2, 4 and 6 h after the carrageenan injection. Oedema was expressed as the increase in paw thickness (in mm) measured after carrageenan injection and compared to the preinjection value for individual animals. Values are expressed as percent inhibition of oedema of treated animals with respect to the carrageenan control group (14).

*Acetic acid-induced vascular permeability*

The comparison between ground licorice and spaceflight group on vascular permeability of Evan’s blue was investigated by Whittle’s method (15). Mice were divided into 14 groups (10/group). Group 1 animals (control) received the normal saline whereas groups 2 were treated with dexamethasone (DEX, 10 mg/kg). Group 3 to 5 animals received sample extract A (1, 2, 4 g/kg); group 6 to 8 animals received sample extract ACK (1, 2, 4 g/kg); group 9 to 11 animals received sample extract H (1, 2, 4 g/kg); group 12 to 14 animals received sample extract HCK (1, 2, 4 g/kg), successive administration were used for 5 days. At the fifth day, 30 min after the i.g. administration, the mice was received an IV injection of 1% Evan’s blue solution (0.1 mL/10 g) to the tail. Ten minutes later, an IP injection of 0.7% acetic acid solution in saline (0.1 mL/10 g) was given to each mouse for the increase of vascular permeability. Twenty minutes after IP administration of acetic acid, the mice were killed. An IP injection of the physiological saline (5 mL each) to wash the inside of the abdominal cavity, after 3 min for kneading the abdomen, collected the eluate, and centrifuged at 1000 r/min for 5 min. The concentration of Evan’s blue in the fluid of the peritoneal cavity was measured by absorbance at 590 nm with a spectrophotometer.

*Statistical methods*

All experimental groups were composed of 10 animals. Data are expressed as mean ± standard deviation Analyses were performed using Student’s t-test and repeated measures one-way analysis of variance (ANOVA) with computer software SPSS 13.0. The values were considered to be significantly different as p < 0.05.

## Results and Discussion

*Components analysis of spaceflight licorice by HPLC*

**Figure 1:**
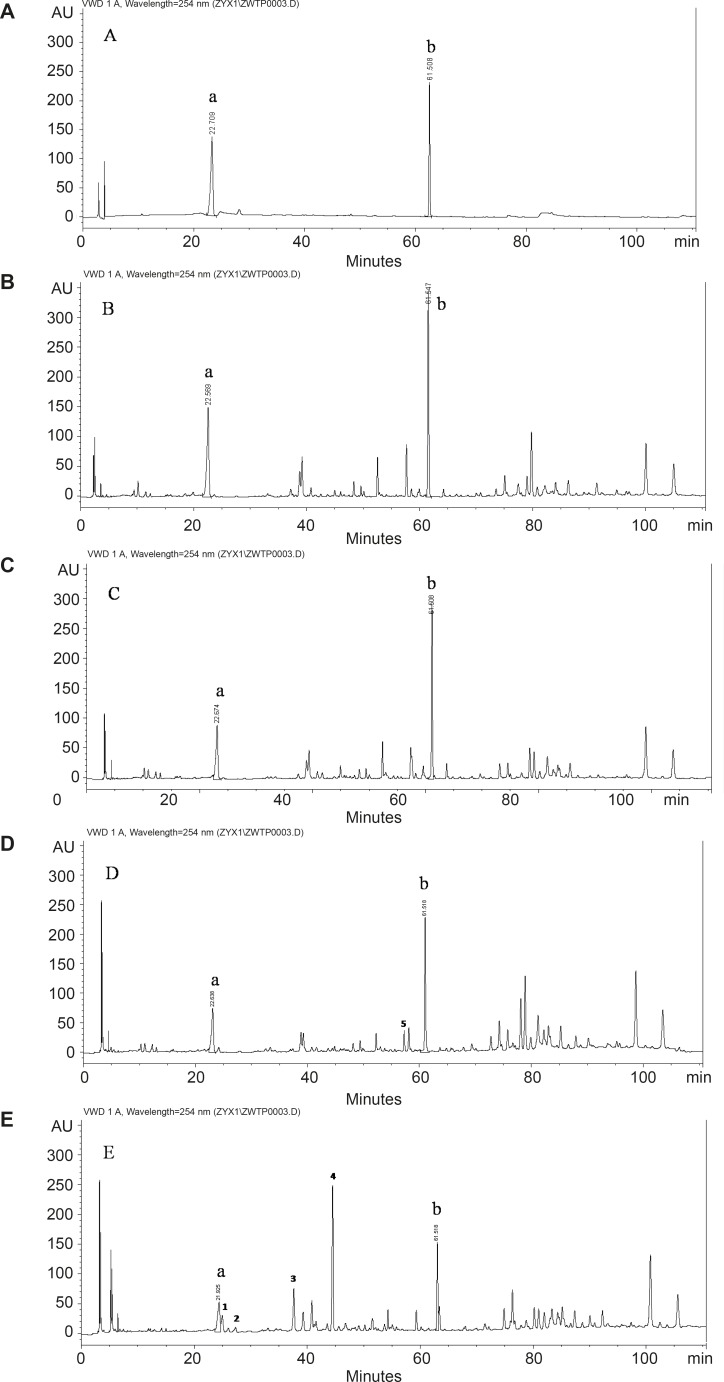
Chromatograms of the components at 254 nm in licorice Standards (B)The group of H (C) The group of HCK (D) The group of A (E) The group of ACK. Peak a: liquiritin; Peak b: glycyrrhizic acid. Peal 1-5: the obvious difference in constituents between the sample A and ACK.

To study the influence of space flight on the components of licorice, chromatographic fingerprint with good resolution of adjacent peaks was obtained. The chromatogram acquired by HPLC-DAD at 254 nm was showed in Figure 1. The differences of main components were found in the groups of H to HCK and A to ACK. 

As for the known constituents, two peaks at retention time of 22.7 min and 61.5 min were identified based on comparison of the standard sample, which were liquiritin and glycyrrhizic acid. The results summarized in Table 1 displayed the contents of liquiritin and glycyrrhizic acid of each group. Comparing the contents of liquiritin and glycyrrhizic acid between the groups of spaceflight and ground control, it increased obviously in the root samples from the licorice seed orbited in an extraterrestrial environment. Especially the content of glycyrrhizic acid in the group of H was two times higher than that of the ground control with significant differences. As for the unknown components, comparison of the fingerprints, there were some differences between the group A and ACK, however, they were not found in H and HCK. As shown in the chromatogram, the conspicuous discrepancy was the peaks 1-5 in the chromatogram. The deep study on the unknown peak should be carried on.

*The effect of space flight on anti-inflammatory activity in licorice roots*

Licorice is the most frequently prescribed plant in traditional Chinese medicine. The extract of licorice roots can inhibit the inflammatory model characterized by increased capillary permeability, swelling or the chronic model characterized by hyperplasia of granulation. The anti-inflammatory activities were compared between the spaceflight group and the ground control one in the two species through three kinds of acute inflammatory model. Xylene-induced ear edema is a well-accepted and popular model for evaluating anti-inﬂammatory activity. As shown in Table 2**,** comparison of the oedema index and inhibition rate in the two species displayed that anti-inﬂammatory activity of samples which carried from the spaceflight were significantly higher than that of the ground control ones in high dose and the diversity was the most obvious in the group of H. 

**Table 1:** Contents (mg g^-1^) of liquiritin and glycyrrhizic acid in licorice root (n = 3).

**Samples**	**Content**	
	**Liquiritin (mg g** ^-1^ **)**	**Glycyrrhizic acid (mg g** ^-1^ **)**
ACK	5.76 ± 0.07	1.91 ± 0.09
A	6.17 ± 0.06^*^	3.66 ± 0.06 ^**^
HCK	6.09 ± 0.05	2.32 ± 0.13
H	6.97 ± 0.05^*^	5.62 ± 0.19^**^

ACK represents the ground control roots obtained from Ordos licorice seeds in Inner Mongolia. HCK represents the ground control samples obtained from Hangjinqi licorice seeds in Inner Mongolia. Sample A and H represent the spaceflight group of ACK and HCK respectively.

Each value represents mean ± standard error of 3 replicates.

The spaceflight groups (A and H) compared with the ground control groups ( ACK and HCK) respectively * p<0.05, ** p<0.01.

The results in Table 3 showed that the extract of licorice roots (0.5, 1, and 2 g/Kg) reduced the carrageenan-induced paw edema durably for 6 h. The subplantar injection of carrageenan in control rats induced an increase in paw thickness over 6 h. At 0.5, 1, 2, and 6 h after *i.g.* administration, pre-treament with licorice root extracts (0.5, 1, and 2 g/Kg) inhibited oedema in a dose-dependent fashion and dexamethasone significantly (p < 0.01) inhibited the increase in paw thickness induced by carrageenan. Vascular permeability induced by acetic acid was measured by Evan’s blue dye from the abdominal cavity. As shown in Table 4, licorice extractions inhibited the acetic acid-induced extravasation of Evan’s blue dye in mice approximately from 9.62% to 37.19% respectively as compared to a control (p < 0.05 and p < 0.01). Thus, it may have a membrane stabilizing effect that reduces the capillary permeability. The comparison of effects between the spaceflight group and ground control samples, it can be displayed that after the spaceflight, the anti-inflammatory activity was higher significantly. All models gained the tendency that the spaceflight group of species H had the strongest activity than other groups.

**Table 2:** Comparison of the effects on auricular edema induced by xylene in mice (*x±s*).

**Group**	***n***	**Dose** **(g/kg)**	**Oedema index (mg)**	**Inhibition** **(%)**
**Control**	10	10 ml	17.06 ± 4.69	—
**DEX**	10	0.01	8.72 ± 3.83^**^	53.86
**ACK**	10	1	16.25 ± 2.94	8.50
**ACK**	10	2	14.67 ± 3.42^*^	17.40
**ACK**	10	4	13.51 ± 3.79^*^	23.93
**A**	10	1	15.88 ± 5.21	10.59^ a^
**A**	10	2	13.79 ± 3.39^*^	22.35^ b^
**A**	10	4	12.82 ± 4.47^*^	27.82^ a^
**HCK**	10	1	16.01 ± 3.27	9.88
**HCK**	10	2	14.99 ± 4.00^*^	15.60
**HCK**	10	4	13.66 ± 4.12^*^	26.46
**H**	10	1	14.40 ± 5.04^*^	18.92^ a^
**H**	10	2	13.07 ± 4.16^*^	26.41^ b^
**H**	10	4	11.50 ± 3.15^**^	35.25^ b^

Compared with control negative groups ^* ^p < 0.05, ^**^ p < 0.01. Compared the spaceflight licorice with the ground control samples ^a^ p < 0.05, ^b^ p < 0.01.

The complex space environment such as cosmic rays, gravity, micro gravity, super vacuum, weak electromagnetic fields and other special factors can cause the changes in morphology and at cytogenetic, physiological and molecular levels (16, 17). Spaceflight induced a higher percentage of cells with chromosomal aberrations (18). The cytogenetic damage and hereditary mutation in plant seeds such as tomatoes, potatoes and fungi have been reported and the results of the space experiments showed that the spaceflight will generate mutation leading to the plant genetic variation (19, 20). Quite a few researches on the space, the environment influence on plant development and metabolism were conducted in order to get new varieties plants of high-quality productive such as germination rate getting higher in wheat after the spaceflight and leaf chlorophyll content increasing in tomatoes and cucumbers. Space science provides a novel and unique opportunity for plant breeding.

**Table 3:** Comparison of the effects on the paw edema induced by carrageenan in rats (*x±s*).

**Group**	***n***	**Dose(g/Kg)**	**Oedema index (%)**				
			**30 min**	**1 h**	**2 h**	**4 h**	**6 h**
**Control**	10	10 mL	66.23 ± 3.34	57.99 ± 5.34	43.80 ± 5.38	36.96 ± 5.80	30.87 ± 3.32
**DEX**	10	0.01	44.62 ± 2.10^**^	28.72 ± 3.19^**^	24.27 ± 1.75^**^	20.95 ± 4.84^**^	10.71 ± 2.99^**^
**ACK**	10	0.5	67.55 ± 1.88	57.51 ± 2.92	43.57 ± 2.22	35.59 ± 0.94	28.27 ± 1.58^*^
**ACK**	10	1	65.59 ± 1.06	56.51 ± 0.52	42.41 ± 2.55	33.49 ± 2.30^*^	25.56 ± 1.86^**^
**ACK**	10	2	64.52 ± 1.19	54.95 ± 2.93^*^	40.26 ± 2.55^*^	30.79 ± 2.30^**^	24.12 ± 1.86^**^
**A**	10	0.5	66.31 ± 1.83	56.43 ± 3.04	41.95 ± 1.51^b^	32.31 ± 1.25^*^^ b^	24.62 ± 2.20^**^^b^
**A**	10	1	62.29 ± 1.91^* a^	55.87 ± 2.93^*a^	39.15 ± 2.56^*^^b^	31.22 ± 1.22^*^^ b^	23.67 ± 1.30^**^^b^
**A**	10	2	58.33 ± 2.12^**^^b^	52.78 ± 1.20^**^^b^	38.33 ± 1.41^**^^b^	26.48 ± 1.68^**^^b^	23.08 ± 0.85^**^^a^
**HCK**	10	0.5	65.18 ± 1.93	57.48 ± 1.04	42.45 ± 1.34	30.11 ± 0.91^*^	25.22 ± 1.12^**^
**HCK**	10	1	64.22 ± 1.09	55.20 ± 1.56^*^	40.25 ± 1.02^*^	25.56 ± 0.67^**^	23.52 ± 0.89^**^
**HCK**	10	2	63.10 ± 1.07^*^	53.71 ± 0.87^**^	39.06 ± 0.79^**^	24.19 ± 0.78^**^	21.30 ± 1.12^**^
**H**	10	0.5	62.24 ± 0.86^**^^b^	54.55 ± 0.70^**^^b^	40.75 ± 1.37^**^^b^	26.80 ± 1.37^**^^b^	24.57 ± 1.10^**^^b^
**H**	10	1	63.47 ± 1.01^*^^ a^	53.52 ± 1.26^**^^b^	39.09 ± 0.52^**^^b^	26.57 ± 1.03^**^^b^	22.47 ± 2.85^**^^b^
**H**	10	2	60.63 ± 0.67^**^^b^	51.55 ± 1.66^**^^b^	37.80 ± 2.00^**^^b^	23.47 ± 1.13^**^^b^	17.40 ± 0.79^**^^b^

Compared with control groups ^* ^p < 0.05, ^**^ p < 0.01. Compared the spaceflight licorice with the ground control samples ^a^ p < 0.05, ^b^ p < 0.01.

As for the medicinal plants, the spaceflight experiments revealed the influences not only focusing on the growth process of plants but also on cell mutations and chromosome changes in plant genomes family. The changes of some enzymes in the plants will lead to the chemical constituents of medicinal plants and finally, affect the efficacy. Gao *et al* investigated the effect of spaceflight on the medicinal plants, *Platycodon grandiflorus*, and *Agastache rugosa* utilizing an electron microscope. The experiments indicated that the plants’ ultrastructure changed after the spaceflight (21, 22).

In our former studies, spaceflight exposure increased the isoperoxidase activity and soluble protein content in the space licorice comparing with the ground control one, as well as the generation of repeatable polymorphic bands. It was demonstrated that weightlessness alone could somewhat trigger genomic alterations and result in increased gene expression, while ionizing radiation would enhance the effect of weightlessness. As two important components in licorice root, liquiritin and glycyrrhizic acid play significant role not only in pharmacodynamic action but in the quantity control. In this study, the HPLC analysis showed that the contents of liquiritin and glycyrrhizic acid in licorice roots were higher in the spaceflight group than that of the ground control group. Licorice is commonly used in medicine and edible plants and anti-inflammatory activity is one of the most important pharmacological activities. In particular, ammonium glycyrrhizate and sodium glycyrrhizate are capable of suppressing the formalin-induced edema in both intact and adrenalectomized animals. The effects of glycyrrhizic acid and its aglycon (glycyrrhetic acid) observed on the model of formalin arthritis in rats was similar to the action of hydrocortisone, while the effects of the former triterpenoids on the model of carrageenan-induced inflammation were less pronounced as compared to the hydrocortisone action. At this point, the comparison of anti-inflammatory activity between the spaceflight licorice and the ground control one was conducted through three kinds of inflammatory models and evaluated the changes in pharmacological activity after the spaceflight. The experimental results showed that the anti-inflammatory activity of the space group in two species was higher than that of the ground control group. The reason for such a discrepancy is not clear but diversities of the bioactive components in individual products may affect the pharmacological activity.

**Table 4:** Comparison of the effects on amount of dye leakage in acetic acid-induced vascular permeability in mice (*x±s*).

**Group**	***n***	**Dose (g/Kg)**	**A**	**Inhibition (%)**
**Control**	10	10 mL	1.521 ± 0.209	—
**DEX**	10	0.01	0.733 ± 0.102^**^	51.80
**ACK**	10	1	1.368 ± 0.197	10.09
**ACK**	10	2	1.303 ± 0.185	14.36
**ACK**	10	4	1.084 ± 0.162^**^	28.85
**A**	10	1	1.375 ± 0.194	9.62
**A**	10	2	1.274 ± 0.177^*^	16.25^a^
**A**	10	4	1.082 ± 0.155^**^	28.93
**HCK**	10	1	1.320 ± 0.184	13.22
**HCK**	10	2	1.191 ± 0.171^*^	21.77
**HCK**	10	4	1.016 ± 0.145^**^	33.23
**H**	10	1	1.326 ± 0.183	12.80
**H**	10	2	1.148 ± 0.152^*^	24.5^b^
**H**	10	4	0.955 ± 0.128^**^	37.19^b^

(A) Compared with control groups ^* ^p < 0.05, ^**^ p < 0.01; (B) Compared the spaceflight licorice with the ground control samples ^a^ p < 0.05, ^b^ p < 0.01.

In the summary, the licorice seeds were carried by a recoverable satellite and subsequently planted. The comparisons were conducted including the main components and anti-inflammation activity between the spaceflight licorice and the ground control samples. The result of HPLC analysis showed the components in licorice changing after the spaceflight. Licorice roots extract were administrated orally to rats; three acute inflammatory models demonstrated the diversity of pharmacological activity after the spaceflight. The anti-inflammatory activity was stronger significantly in space group than in ground control one. Therefore, it may be concluded that the space mutagenesis breeding is an efficient approach to the medicinal plant breeding.
